# Field Emission Scanning Electron Microscope Analysis of the Marginal Adaptation of Various Root Canal Sealers at the Dentin-Sealer and Sealer-Guttapercha Interfaces at Three Root Canal Levels: An In Vitro Study

**DOI:** 10.7759/cureus.66156

**Published:** 2024-08-05

**Authors:** Srikumar GPV, Megha Ghosh, Rupashree Chatterjee, Aarushi Gajpal, Mohammed Mustafa, Ahmed A Almokhatieb

**Affiliations:** 1 Department of Conservative Dentistry and Endodontics, Triveni Institute of Dental Sciences, Hospital and Research Centre, Bilaspur, IND; 2 Department of Pediatric and Preventive Dentistry, Triveni Institute of Dental Sciences, Hospital and Research Centre, Bilaspur, IND; 3 Department of Conservative Dental Sciences, College of Dentistry, Prince Sattam Bin Abdulaziz University, Al-Kharj, SAU; 4 Department of Conservative Dentistry and Endodontics, Center for Transdisciplinary Research, Saveetha Dental College, Saveetha Institute of Medical and Technical Sciences, Saveetha University, Chennai, IND

**Keywords:** field emission scanning electron microscope, sealer-guttapercha interface, sealer-dentin interface, marginal adaptation, interfacial gaps

## Abstract

Aims

An in vitro evaluation of the marginal adaptation of four root canal sealer variants at the dentin-sealer and sealer gutta-percha interfaces in the coronal, middle, and apical thirds of root canals was conducted using field emission scanning electron microscope (FESEM) analysis.

Materials and methods

In total, 80 extracted human mandibular premolar teeth were used in this study. All teeth were decoronated to standardize the root length to 14 mm. A round bur was used to gain access into canal orifices and the working length was determined. Root canal instrumentation was performed using the crown-down technique with ProTaper Next rotary files up to size X3, along with the use of root canal irrigants.

All specimens were then randomly divided into four groups, with 20 specimens per group, depending on the root canal sealer used: Group A consisted of AH Plus (Dentsply Maillefer, Ballaigues, Switzerland), Group B of MTA-Fillapex (Angelus Dental, Londrina, Brazil), Group C of Bio-C Sealer (Angelus Dental), and Group D of GuttaFlow-2 (Coltene Whaledent, Altstatten, Switzerland).

All specimens were obturated with size X3 gutta-percha points uniformly coated with respective sealers in the single-cone technique and coronal access was sealed with glass ionomer cement. The specimens were incubated for seven days and then horizontally sectioned at the coronal, middle, and apical thirds of the root canals. On each sample obtained, three points were randomly chosen and both sealer-dentin and sealer gutta-percha interfaces were examined under FESEM at 1000x magnification. The marginal gaps of all four sealers at both interfaces and at three levels of root canals were measured in µm and values were recorded, tabulated, and used for data analysis. One-way analysis of variance (ANOVA) and post hoc Bonferroni tests were used for statistical analysis. A p-value ≤ 0.05 was considered statistically significant.

Results

Compared to the sealer gutta-percha interface, AH Plus, MTA-Fillapex, and Bio-C sealers at all three levels of the root canals showed more marginal gaps at the sealer-dentin interface with a significant difference (p<0.05). However, the GuttaFlow-2 sealer showed no significant difference (P > 0.05).

Conclusions

The marginal adaptation of the GuttaFlow-2 sealer is superior to both dentin and gutta-percha at the coronal, middle, and apical thirds of root canals compared to other sealers used in the study.

## Introduction

The objective of endodontic therapy is to eliminate diseased root canal tissue and its contents to rectify periapical infection and inflammation. The breakdown of periapical tissues can be halted only when the root canal system is sealed from the periodontal ligament and surrounding bone; this can be achieved with the use of instruments and antiseptics, followed by three-dimensional filling or obturation of root canal space 0.5 mm to 1 mm from the radiographic apex [1]. Ideal obturation involves thoroughly filling the entire dentinal portion of the root canals, effectively sealing the cemento-dentinal junction, and stimulating the obliteration of the cemental portion of the canal with new cementum deposition [2].

Several materials were tried and tested as endodontic obturation materials, of which gutta-percha has been most extensively used for years worldwide and has established itself as a gold standard [3]. The composition of gutta-percha cones used for root canal obturation is 56-66% zinc oxide fillers, 20% gutta-percha, 11% barium sulfate, and 3% waxes [4]. Gutta-percha alone cannot seal the root canal space, as it has no adhesion and only adapts to the root canal dentin. Root canal sealers are usually regarded as the seal-forming 'gasket' of the root canal filling but can also act as a weak link in the root canal space; thus, their volume should be minimized with more gutta-percha cones used as core filling material [5]. The rationale for the use of a sealer is to attain a fluid-tight seal apically, laterally, and coronally between the root canal dentin and gutta-percha. Root canal sealer acts as a lubricating agent, helping in the proper seating of gutta-percha cones in root canals and also as a binding agent between the gutta-percha cones [6].

The ability of root canal sealers to penetrate deeper into the dentinal tubules consistently and effectively is one of the many factors influencing the choice of the sealer used in root canal obturation [7]. Four varieties of root sealer were used in this study, which are described below:

AH Plus (Dentsply Maillefer, Ballaigues, Switzerland) root canal sealer is available in a two-paste system. The epoxide paste includes radiopaque fillers and aerosol, while the amino paste consists of 1-adamantane amine, N, N-dibenzyl-5-oxanonandiamine-1,9, and TCD diamine [8]. MTA Fillapex (Angelus Dental, Londrina, Brazil) is a calcium silicate-based root canal sealer composed of salicylate resin, diluting resin, natural resin, bismuth trioxide, nano-particulate silica, MTA (tricalcium silicate, dicalcium silicate, tricalcium aluminate, tetra calcium alumino-ferrite-silicate) and pigments [9]. Bio-C Sealer (Angelus Dental) is a new premixed bioceramic-based root canal sealer available in a single syringe form and is composed of calcium silicates, calcium aluminate, calcium oxide, zirconium oxide, iron oxide, silicon dioxide, and dispersing agents [10]. GuttaFlow 2 (Coltene Whaledent, Altstatten, Switzerland) is a silicone-based root canal sealer available in Automix syringe form. It contains gutta-percha powder, polydimethylsiloxane, platinum catalyst, zirconium dioxide, and micro-silver [11].

Early models of scanning electron microscopes (SEM) used a heated tungsten filament cathode as an electron source, known as a thermionic emitter. Later in 1975, lanthanum hexaboride cathodes replaced tungsten cathodes. Thermionic emitters emit high current with a beam size of 4 to 8 nm, but they produce low brightness and also result in the evaporation of filament, called thermal drift, limiting optical performance at higher resolutions. A field emission scanning electron microscope (FESEM) is a type of SEM that utilizes a field emitter gun (FEG) as its emitter type. FESEM produces a higher electron density with a beam size focus of 2 nm, resulting in a higher resolution than SEM. The electromagnetic lenses and apertures of FESEM focus the electron beam it produces into a tiny, sharp spot that is about 1000 times smaller than in SEM. This results in improved image quality, enabling the examination of minute features, demarcations, the presence of gaps and voids, and the adaptation of any two material surfaces more clearly [12].

Hence, this in-vitro study aimed to evaluate the marginal adaptation of AH Plus, MTA Fillapex, Bio-C, and GuttaFlow-2 root canal sealers at the dentin-sealer and sealer gutta-percha interfaces in the coronal-third, middle-third, and apical-third of root canals using FESEM analysis.

## Materials and methods

An in vitro study was conducted in the after obtaining the Institutional Ethical Committee clearance certificate, TIDSHRC/IEC/2021/D006, at the Department of Conservative Dentistry and Endodontics, Triveni Institute of Dental Sciences, Hospital and Research Centre, Bilaspur, India. Our study sample consisted of 80 human permanent mandibular single-rooted premolar teeth. The sample size was calculated using the following formula:

$n=2x (Z&alpha;/2+Z&beta;)2x \sigma2/&Delta;2$

Where Z_α/2​_: Z-value corresponds to the desired significance level (α), typically 1.96 for α = 0.05; Z_β_​: Z-value corresponds to the desired power (1-β), typically 0.84 for 80% power, σ: standard deviation of the population, Δ: minimum detectable difference between the means.

The inclusion criteria for this study were human permanent mandibular single-rooted premolar teeth, extracted either for orthodontic reasons or due to periodontal compromise, from individuals aged 18-30 years. The selected teeth had to be intact, without any caries or non-caries lesions, and free from prior restorations, clinically detectable fractures, or cracks. In total, 80 teeth were chosen. Each tooth was examined under a stereomicroscope (Olympus SZ61, Olympus Optical Co., Tokyo, Japan) at 10X magnification to ensure they met these conditions and had no open root apices. Digital periapical radiographs using radiovisiography (RVG) (Carestream Health India Pvt. Ltd., Mumbai, India) were then taken in both buccolingual and mesiodistal directions to confirm the presence of a single root canal. Additionally, the root canal curvature had to be less than 20º, as determined by Schneider’s criteria [13].

The collected teeth were cleaned of superficial debris, calculus, and residual tissue tags using ultrasonic instruments and were then stored in 0.5% thymol at room temperature until used. Occupational Safety and Health Administration (OSHA) and Centre for Disease Control (CDC) recommendations and guidelines were strictly followed during the collection, sterilization, and handling of extracted teeth to prevent any biohazard transmission. All specimens were then decoronated using a flexible diamond disc attached to a straight micromotor handpiece at a low speed under a water coolant at the level of the cementoenamel junction perpendicular to the long axis of teeth; this was done to standardize the root length to 14 mm, which was measured by a Vernier calliper. A no. 4 round bur was attached to a contra-angle high-speed air rotor handpiece to gain access to the root canal orifices of all specimens. A no. 10 K-file was placed into the root canal to establish the patency till the apical foramen, and with the tip of the trail file just visible at the apical foramen, the working length was determined by subtracting 0.5 mm from the length in all specimens.

All specimens were then numbered randomly from 1 to 80, with the numbers written on the coronal third of roots of each specimen using a permanent marker pen, and were then individually embedded in cylindrical self-cure acrylic resin blocks up to the level 3 mm apical to the cemento-enamel junction to facilitate ease of handling during root canal instrumentation.

Root canal instrumentation was performed in all specimens in crown-down technique using the ProTaper Next rotary file system in file sequences of X1, X2, and X3, with X3 as the Master Apical File (MAF) (size 0.30, taper 0.07) at 300 rpm, with 2.8 Ncm of torque attached to a torque-controlled endomotor handpiece. Each rotary file was used for instrumentation in only five root canals and was discarded, followed by the use of new files. In each root canal, during instrumentation, 2 ml of 17% EDTA solution (ethylene diamine tetraacetic acid) and 2 ml of 3% sodium hypochlorite solution were used as root canal irrigants with Max-I-Probe irrigation needles and each root canal was then rinsed with 2 ml of distilled water to remove any remnants of root canal irrigants.

All specimens (n = 80) were then randomly divided into four groups, with 20 specimens per group depending upon the root canal sealer used; they were then obturated with gutta-percha in the single-cone technique described below:

In Group A (n = 20) and Group B (n = 20), root canals were completely dried with sterile absorbent paper points, and AH Plus and MTA Fillapex sealers were manipulated on paper pads consistently as per the manufacturer's instructions, respectively. ProTaper Next gutta-percha points, Size X3 (Dentsply, Maillefer, Switzerland), were then uniformly coated with the respective sealers and slowly inserted into the root canals until they reached the predetermined working length and were checked for tug-back.

In Group C (n = 20), the Bio-C Sealer is premixed, so it was directly dispensed on a paper pad, as per manufacturer instructions. Root canals were not completely dried with sterile absorbent paper points; instead, they were left slightly moist. ProTaper Next gutta-percha points, size X3, were then uniformly coated with the sealer and slowly inserted into the root canals until they reached the predetermined working length and were checked for tug-back.

In Group D (n = 20), root canals were dried with sterile absorbent paper points and GuttaFlow-2 sealer (Coltene Whaledent Pvt. Ltd., Altstatten, Switzerland) was manipulated on a paper pad following the manufacturer's instructions. ProTaper Next gutta-percha points, Size X3, were then uniformly coated with the sealer and slowly inserted into root canals until they reached the predetermined working length after which they were checked for tug-back.

In all specimens, gutta-percha was seared off at the level of the cemento-enamel junction using a heated plugger and vertically compacted 2 mm below the cementoenamel junction and the coronal access of root canals was sealed with Light-Cure Glass Ionomer Cement. To prevent any inter-operator variability, the root canal instrumentation and obturation in all specimens were conducted by a single endodontist. Later, to allow adequate time for the complete setting of all four root canal sealers, all specimens were carefully removed from the acrylic resin blocks and placed in an incubator at 100% relative humidity and 37 °C for seven days.

All specimens were then horizontally sectioned using a flexible diamond disc attached to a straight micromotor handpiece at low speed under continuous water flow to prevent any frictional heat generation and minimize smearing of gutta-percha and root canal sealer to obtain 1 mm thick cross-sections of roots at 3 mm (apical-third), 6 mm (middle-third), and 9 mm (coronal-third) from the predetermined working length. For ease of identification, distinctive labelling was done to all the cross-sections obtained from each specimen of Groups B, C, and D to differentiate them as apical-third, middle-third, and coronal-third samples. The samples were then examined under a FESEM (Quanta FEG 250, Thermo Fisher Scientific, Oregon) at a magnification of 1000X. Three randomly chosen points in the coronal-third, middle-third, and apical-third cross-sections of each root specimen were focused under FESEM both at the dentin-sealer and sealer gutta-percha interfaces. The marginal adaptation was measured using Image J software (National Institute of Mental Health, Bethesda, MD) and calculated at both interfaces, after which the mean (µm) was estimated for each sample. To prevent any inter-operator variability, FESEM photomicrographs of all specimens were analysed by a single endodontist. The reliability of measurements was ensured through intra-rater reliability testing, wherein the same endodontist re-evaluated a subset of samples (20% of the total) after a two-week interval. The intraclass correlation coefficient (ICC) was calculated to assess the consistency of measurements, with an ICC value of 0.95 indicating excellent reliability. The obtained data was recorded and tabulated.

IBM SPSS software v. 24 (IBM Corp., Armonk, NY), was used for data analysis. One-way ANOVA (analysis of variance) was used to compare the extent of marginal adaptation at dentin-sealer and sealer-gutta-percha interfaces among the four experimental groups at three root levels. A post hoc Bonferroni test was done for pairwise comparison between the experimental groups. A p-value ≤ 0.05 was considered statistically significant.

## Results

A one-way ANOVA test between the four groups for the presence of marginal adaptation both at dentin-sealer and sealer-gutta-percha interfaces showed Group A (AH Plus sealer) with maximum marginal adaptation and Group D (GuttaFlow-2 sealer) with least marginal adaptation at the coronal-third, middle-third and apical-third of root canals. A statistically significant difference was seen in the marginal adaptation expressed in µm between the four groups tested (P≤0.05), summarized in Tables 1-2.

**Table 1:** One-way ANOVA test for inter-group comparison of marginal adaptation at dentin-sealer interface of the specimens from Groups A-D P: probability, SD: standard deviation

Root canal levels	Group A (AH Plus sealer)	Group B (MTA Fillapex Sealer)	Group C (Bio-C Sealer)	Group D (GuttaFlow-2 Sealer)	p-value
Coronal third	7.68 ± 5.47	8.58 ± 6.06	7.22 ± 5.14	6.34 ± 3.68	0.58
Middle third	12.69 ± 5.68	8.13 ± 5.34	7.79 ± 5.1	1.94 ± 1.39	0.001
Apical third	6.12 ± 5.45	4.82 ± 3.93	5.75 ± 5.98	2.35 ± 1.26	0.04

**Table 2:** One-way ANOVA test for inter-group comparison of marginal adaptation at sealer gutta-percha interface of the specimens from Groups A-D P: probability, SD: standard deviation

Root canal levels	Group A (AH Plus Sealer)	Group B (MTA Fillapex Sealer)	Group C (Bio-C Sealer)	Group D (GuttaFlow-2 Sealer)	p-value
Coronal-third	42.80 ± 22.69	2.84 ± 2.52	3.21 ± 2.28	0.60 ± 0.85	0.001
Middle- third	11.99 ± 17.28	1.33 ± 1.10	1.84 ± 1.27	0.22 ± 0.49	0.001
Apical- third	9.27 ± 19.73	3.18 ± 2.59	2.63 ± 2.40	0.09 ± 0.17	0.03

At the dentin-sealer interface, Group A (AH Plus) showed maximum marginal adaptation at the middle third (12.69 µm), followed by the coronal third (7.68 µm) and the apical third (6.12 µm). Group B (MTA Fillapex) showed maximum marginal adaptation at the coronal third (8.58 µm), followed by the middle third (8.13 µm) and the apical third (4.82 µm). Group C (Bio-C) showed maximum marginal adaptation at the middle third (7.79 µm), followed by the coronal third (7.22 µm) and the apical third (5.75 µm). Group D (GuttaFlow-2) showed maximum marginal adaptation at the coronal third (6.34 µm followed by the apical third (2.35 µm) and the middle third (1.94 µm)).

**Figure 1:**
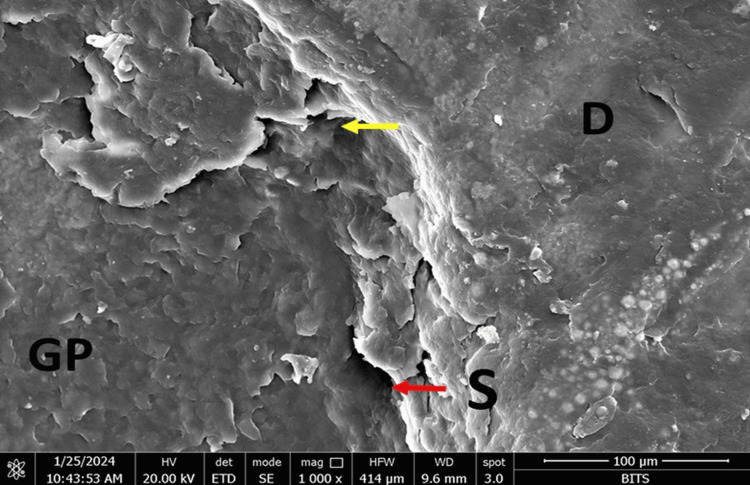
FESEM photomicrograph of a specimen from Group A with AH Plus sealer at the coronal third of the root canal. The red and yellow arrows denote the interface. D: dentin, S: sealer, GP: gutta-percha

**Figure 2:**
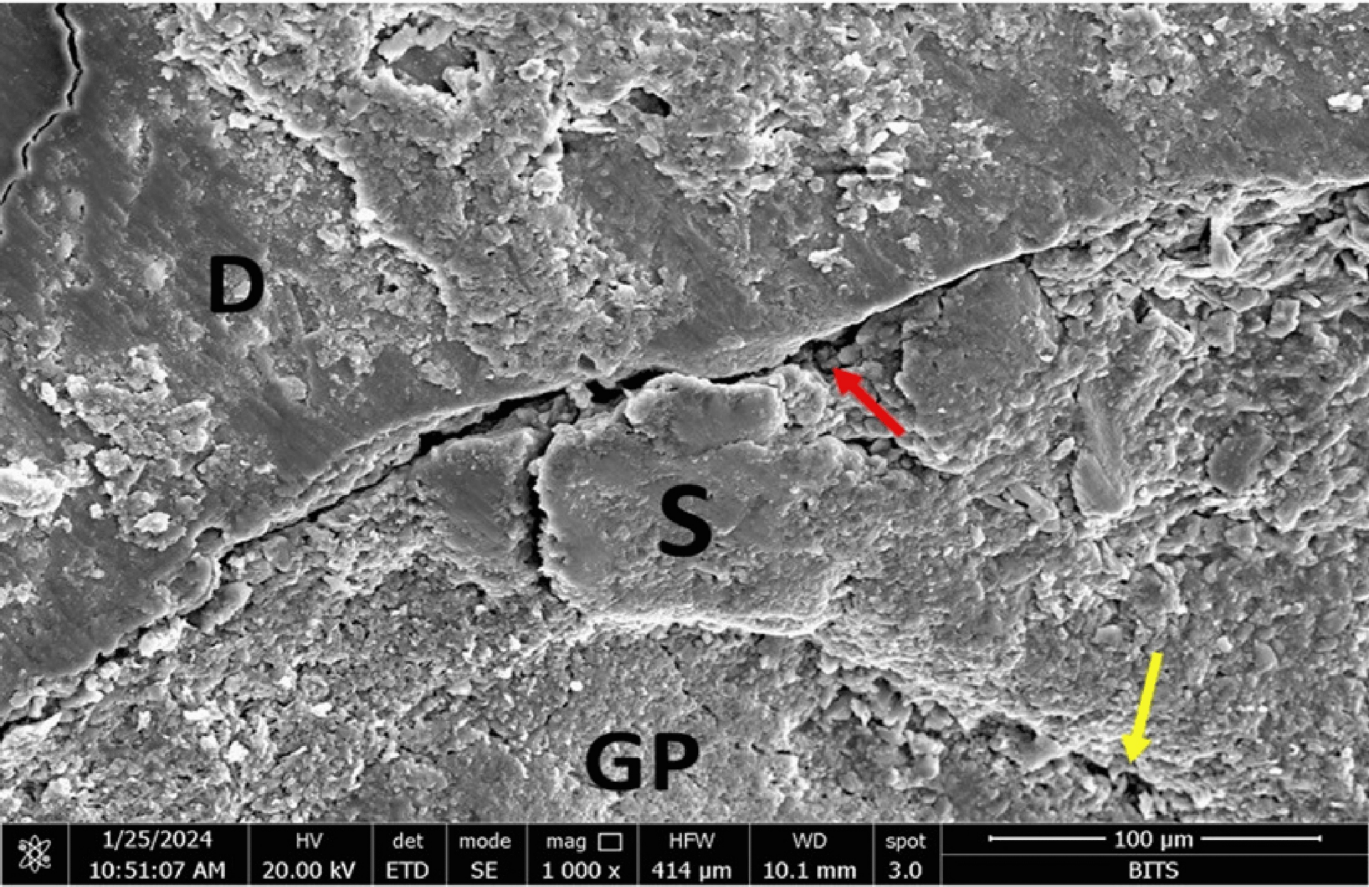
FESEM photomicrograph of specimen of Group B (MTA fillapex sealer) at the apical third of the root canal. The red and yellow arrows denote the interface. D: dentin, S: sealer, GP: gutta-percha

**Figure 3:**
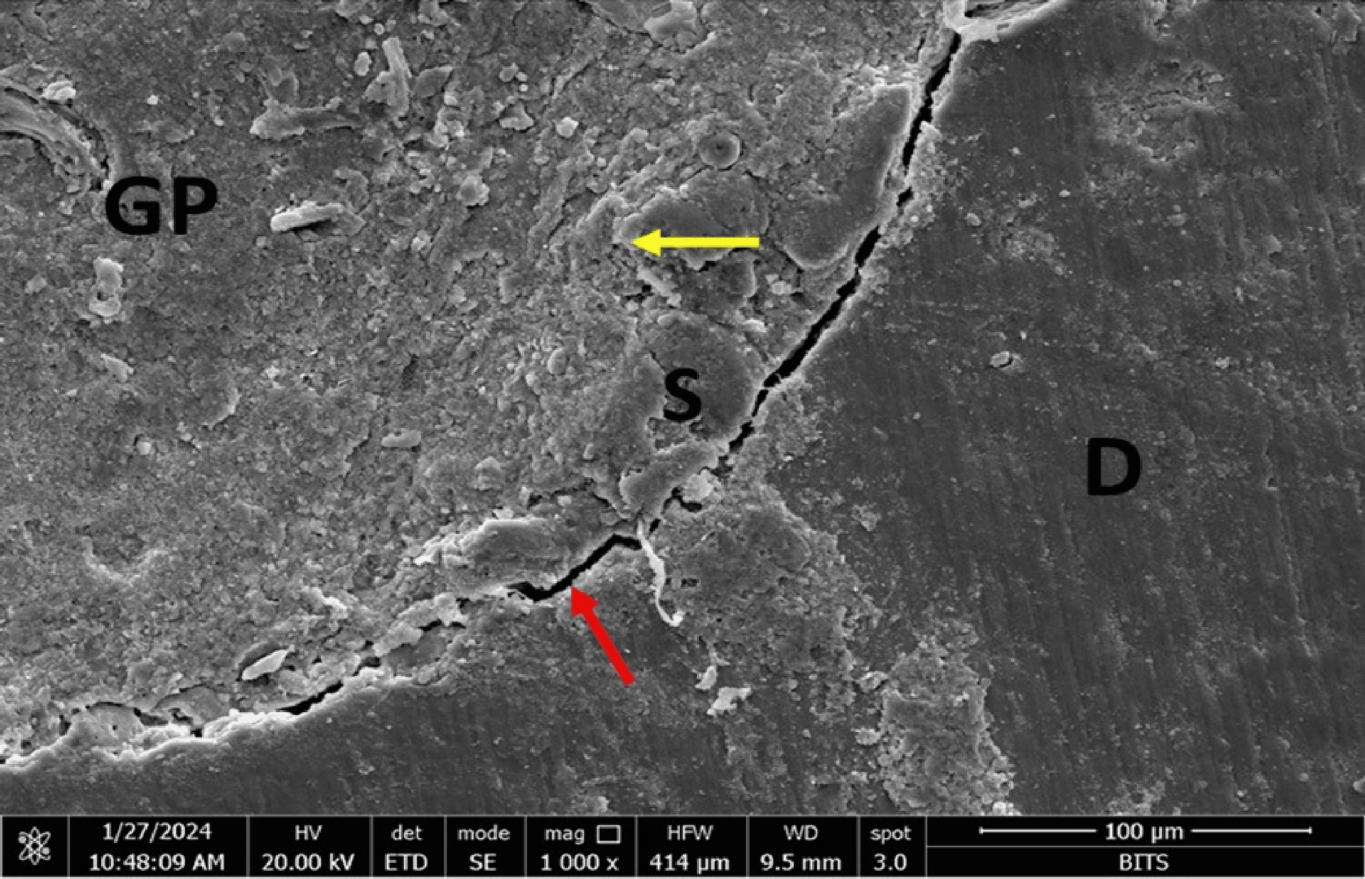
FESEM photomicrograph of a Group C (Bio C sealer) specimen at the apical third of root canal. The red and yellow arrow marks denote the interface. D: dentin, S: sealer, GP: gutta-percha

**Figure 4:**
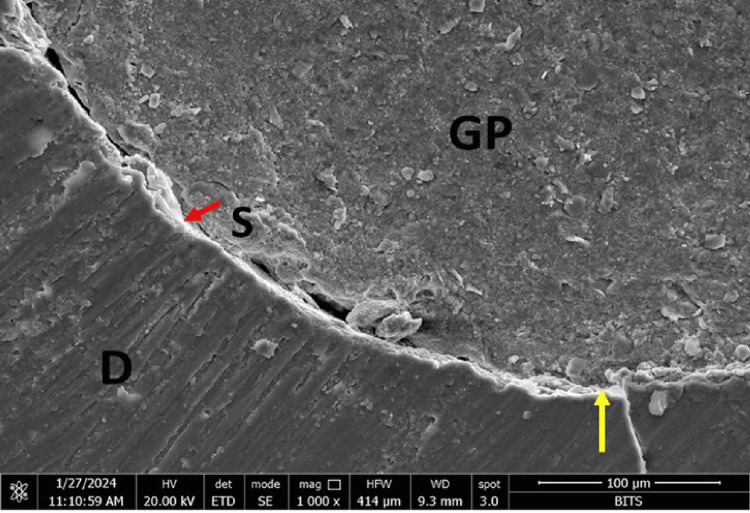
FESEM photomicrograph of a Group D specimen (GuttaFlow 2 sealer) at the coronal third of the root canal. The red and yellow arrows denote the interface. D: dentin, S: sealer, GP: gutta-percha

At the sealer-gutta-percha interface, Group A (AH Plus) showed maximum marginal adaptation at the middle third (11.99µm), followed by the coronal third (6.34 µm) and the apical third (9.27 µm). Group B (MTA Fillapex) showed maximum marginal adaptation at the apical third (3.18 µm), followed by the coronal third (2.84 µm) and the middle third (1.33 µm). Group C (Bio-C) showed maximum marginal adaptation at the coronal third (3.21 followed by the apical third (2.63 µm) and the middle third (1.85µm). Group D (GuttaFlow-2) showed maximum marginal adaptation at the coronal third (0.60 µm followed by the middle third (0.22 µm) and the apical third (0.09 µm) (Figures 1-4).

Post hoc Bonferroni analysis test was done to compare the significance in pair-wise groups for the marginal adaptation at dentin-sealer and sealer gutta-percha interfaces at the coronal third, the middle-third and apical-third of root canals of all specimens (Tables 3-4).

**Table 3:** Post hoc Bonferroni test in pair-wise comparison of marginal adaptation at dentin-sealer interface in coronal, middle and apical thirds of root canals Bonferroni adjustment factor α = 0.008; P < α = Significant, P > α = Non-significant, *: Significant

Pair-wise Comparison	Coronal-third P value	Middle-third P value	Apical-third P value
Group A vs Group B	0.62	0.012	0.39
Group A vs Group C	0.78	0.006*	0.83
Group A vs Group D	0.37	<0.00001*	0.004*
Group B vs Group C	0.44	0.83	0.56
Group B vs Group D	0.16	<0.00001*	0.01
Group C vs Group D	0.54	<0.00001*	0.01

**Table 4:** Post hoc Bonferroni test in pair-wise comparison of marginal adaptation at sealer Gutta-percha interface in coronal, middle and apical thirds of root canals P: Probability; Bonferroni adjustment factor α = 0.008; P < α = Significant, P > α = Non-significant, *: Significant

Pair-wise Comparison	Coronal-third P value	Middle-third P value	Apical-third P value
Group A vs Group B	<0.0001*	0.0089	0.17
Group A vs Group C	<0.0001*	0.012	0.14
Group A vs Group D	<0.0001*	0.004*	0.04
Group B vs Group C	0.63	0.17	0.48
Group B vs Group D	0.0005*	0.0001*	<0.0001*
Group C vs Group D	<0.0001*	<0.0001*	<0.0001*

Post hoc Bonferroni analysis of the dentin-sealer interface showed that at the coronal third, no significant differences were seen in pair-wise comparison among the four groups; at the middle third, statistically significant differences were seen in group pairs of Group A vs Group C, Group A vs Group D, Group B vs Group D and Group C vs Group D; and at the apical third, statistically significant differences were seen in group pairs of Group A vs Group D.

Post hoc Bonferroni analysis of the sealer gutta-percha interface showed that at the coronal third, statistically significant differences were seen in group pairs of Group A vs Group B, Group A vs Group C, Group A vs Group D, Group B vs Group D and Group C vs Group D; at the middle third, statistically significant differences were seen in group pairs of Group A vs Group D, Group B vs Group D, and Group C vs Group D; and at the apical-third level, statistically significant differences were seen in group pairs of Group B vs Group D, Group C vs Group D.

None of the groups showed complete marginal adaptation at both dentin-sealer and sealer gutta-percha interfaces at all three levels of the root canal; there were both gap-free and gap-containing areas at all three levels of the root canal in all groups. However, the GuttaFlow-2 sealer (Group D) exhibited maximum marginal adaptation and AH Plus sealer (Group A) showed minimum marginal adaptation at both dentin-sealer and sealer gutta-percha interfaces at all three levels of the root canal. Compared to the sealer gutta-percha interface, AH Plus, MTA Fillapex, and Bio-C sealers at all three levels of the root canal showed more marginal gaps at the sealer-dentin interface with a significant difference (P<0.05). However, the GuttaFlow-2 sealer showed no significant difference (P > 0.05). 

At the dentin-sealer interface in the coronal third of root canals, AH Plus and MTA Fillapex sealers showed the maximum and GuttaFlow-2 sealers showed the minimum marginal adaptation. In the middle-third and apical-third of root canals, AH Plus sealer showed the maximum and GuttaFlow-2 sealer showed the minimum marginal adaptation.

At the sealer gutta-percha interface, in the coronal-third, middle-third and apical-third of root canals, the AH Plus sealer showed the maximum marginal adaptation and the GuttaFlow-2 sealer showed minimum marginal adaptation.

## Discussion

The main objective of obturation is to provide a three-dimensional seal of root canal space, thus preventing root canal reinfection and preserving the health of periapical tissues. An ideal root canal obturation is to have a greater volume of gutta-percha as the core filling material with a minimal volume of root canal sealer penetrates deeper into the canal irregularities and dentinal tubules [14]. In the present study, single-canal mandibular premolar teeth were utilised because they have approximately similar bucco-lingual and mesio-distal dimensions. The teeth were decoronated at the cementoenamel junction to eliminate variations in endodontic access cavity preparation designs.

As gutta-percha lacks any adhesive properties, a root canal sealer helps to adapt it to the root canal dentin and fills any canal imperfections, thus preventing microleakage, microbial contamination, and failure of endodontic therapy. An ideal root canal sealer should be biocompatible and have low surface tension, thereby having deeper penetration into the dentinal tubules of the root canal and better wettability, providing a fluid-tight seal. About 60% of endodontic failures are due to inadequate filling of the root canal space. Because of the hydrophobic nature of gutta-percha, the sealer tends to pull away from it upon its setting, leading to the formation of microscopic gaps at dentin-sealer and sealer gutta-percha interfaces and the leakage through these marginal adaptations is a major reason for the failure of root canal therapy [15].

The use of the lateral condensation technique of obturation was reported to produce non-homogenous thick layers of sealers along the walls of the root canal, thus influencing the sealer penetration into the root dentin [16]. In the present study, all specimens were obturated with gutta-percha using the single-cone technique, as it is the most commonly used method in clinical situations. With this technique, the volume of the sealer used is minimized, and the use of calibrated X3 gutta-percha points as supplied by the manufacturer for obturation corresponds to the size and taper of the X3 file, thus maintaining homogeneity among the specimens of all four groups. The single-cone obturation technique became very popular with the use of Rotary Ni-Ti (nickel-titanium) file systems for root canal instrumentation, followed by the use of corresponding sizes and tapers of gutta-percha points for obturation. This technique was stated to be time-saving compared to the commonly used lateral condensation technique of obturation [16]. In our study, after root canal obturation, all specimens were incubated at 37 °C for seven days to ensure the setting of sealers was completed.

In the present study, an FESEM was utilized for the assessment of marginal adaptation of the root canal sealers at dentin-sealer and sealer gutta-percha interfaces. FESEM provides topographical and elemental information at a magnification of 10X to 3000,000X with virtually unlimited depth of field. Compared to SEM, FESEM produces clear, less electrostatically distorted images with spatial resolution down to 11/2 nanometers, which is three to six times better. FESEM allows a large amount of the sample to be analyzed at any given time and the final evaluation can be conducted by preserving photomicrographs [17]. In the present study, all four root canal sealers showed marginal gaps at both sealer-dentin and sealer gutta-percha interfaces at the coronal, middle and apical thirds of root canals. However, maximum marginal adaptation was seen at the coronal third, followed by the middle third, and was the least in the apical third levels.

In comparison with the findings of our study, previous literature has highlighted various aspects of root canal sealer performance and marginal adaptation. For instance, studies by Jain et al. have shown that GuttaFlow-2 sealer exhibits superior marginal adaptation at the dentin-sealer and sealer-gutta-percha interfaces compared to other sealers, which is consistent with our observations [18-20]. The unique properties of GuttaFlow-2, such as its ability to expand slightly upon setting, contribute to its enhanced sealing ability and reduced microleakage, thus supporting our results. Moreover, Bouillaguet et al. also confirmed that the combination of gutta-percha particles in a polydimethylsiloxane matrix within GuttaFlow-2 significantly improves the intimate adaptation to the root canal dentin and gutta-percha, corroborating our findings regarding its superior performance in the apical third of root canals [21].

In contrast, the marginal adaptation of AH Plus sealer has been documented to show more considerable gaps at both the dentin-sealer and sealer-gutta-percha interfaces, attributed to its linear setting shrinkage and hydrophobic nature, as noted by previous studies [22,23]. Our study aligns with these findings, demonstrating greater mean marginal gaps with AH Plus compared to other sealers. Additionally, research on MTA Fillapex has indicated that while it can expand during its setting reaction, improving sealing ability, it often exhibits more marginal gaps at the sealer-dentin interface, similar to our observations [24]. Bio-C Sealer, with its hydrophilic properties and chemical bonding capabilities, has been reported to provide better marginal adaptation compared to AH Plus, which our study supports [25]. However, our findings also highlight that Bio-C Sealer showed more marginal gaps at the sealer-dentin interface than at the sealer-gutta-percha interface, suggesting areas for further investigation to optimize its performance. These comparisons with existing literature reinforce the validity of our study's results and provide a broader context for understanding the performance of different root canal sealers.

In the present study, FESEM was used for critical evaluation of genuine gaps at both sealer-dentin and sealer gutta-percha interfaces, as potential artifactual gaps generally happen with vacuum desiccation in conventional SEM studies. Marginal gaps at the dentin-sealer and sealer gutta-percha interfaces may jeopardize the successful outcome of root canal treatment, so a complete seal at both of these two interfaces at all three levels of the root canal is essential.

This study's limitations include the sample size of 80 extracted human mandibular premolar teeth. Though standardized to a root length of 14 mm, they may not fully represent the variability encountered in clinical settings. The single-cone obturation technique employed might not reflect outcomes from other techniques like lateral condensation or thermoplasticized methods. The seven-day incubation period might not suffice to assess the long-term stability and adaptation of the sealers. The controlled laboratory conditions do not account for the myriad of clinical variables such as operator skill and intraoral conditions, which can significantly influence the performance of root canal sealers in practice.

For future research, it is recommended to include a larger and more diverse sample size to better represent clinical variability. Additionally, incorporating various obturation techniques and extending the incubation period could provide more comprehensive insights into the long-term performance of the sealers. Evaluating the sealers under more clinically relevant conditions, including different operator skill levels and intraoral environments, would enhance the applicability of the findings to real-world scenarios.

## Conclusions

Under the conditions of our study, it was found that GuttaFlow-2, a silicone-based root canal sealer, exhibited superior marginal adaptation with the fewest gaps and AH Plus sealer showed poor marginal adaptation at both dentin-sealer and sealer gutta-percha interfaces at all three levels of the root canal. GuttaFlow-2 adapted better to root canal dentin as well as to gutta-percha at all three levels of the root canal, including the most critical apical third level, and thus can be the most effective root canal sealer among the four to be used in clinical practice. Nonetheless, further in vivo studies are needed to confirm and correlate the findings of this in vitro study.

## Appendices

List of instruments used

(i) flexible diamond disc (Novo Dental Products, Mumbai)

(ii) straight micromotor handpiece (NSK Ltd., Tokyo Japan)

(iii) Vernier caliper. A No. 4 Round Bur (Mani Inc., Utsunomiya, Japan)

(iv) contra-angle high-speed airotor handpiece (NSK Ltd.)

(v) A no.10 K-file (Dentsply, Maillefer, Switzerland)

(vi) DPI RR Cold Cure, Dental Product of India Ltd., Mumbai, India)

(vii) ProTaper Next rotary file system (Dentsply, Maillefer, Switzerland) in file sequences of X1, X2 and X3, with X3 as the Master Apical File (MAF) (size 0.30, taper 0.07)

(viii) Torque-controlled endomotor handpiece (CanalPro CL2, Coltene Endo, Coltene Whaledent Pvt. Ltd., Alstatten, Switzerland)

(ix) 17% EDTA (ethyline diamine tetraacetic acid) solution (Prevest DenPro, Bari Brahmana, India)

(x) 3% sodium hypochlorite solution (Neelkaanth Health Care Pvt. Ltd., Ahmedabad, India)

(xi) Max-I-Probe (Dentsply Maillefer)

(xii) Distilled water (Sadbhavna Chemicals, Saktasanala, India)

(xiii) sterile absorbent paper points (DiaDent International, Cheongju, Republic of Korea)

(xiv) sterile absorbent paper points (AH Plus, Dentsply Sirona Global, USA)

(xv) heated plugger (BeeFill Heat Plugger, VDW GmbH, Munich, Germany)

(xvi) Light-Cure Glass Ionomer Cement (GC Light-Cure Universal Restorative, GC Corp., Tokyo, Japan)

(xvii) Incubator (Zeal International, New Delhi, India)
